# Comments to: Risk factors for late defecation and its association with the outcomes of critically ill patients: a retrospective observational study

**DOI:** 10.1186/s40560-016-0170-3

**Published:** 2016-07-19

**Authors:** Dominique Prat, Benjamin Sztrymf

**Affiliations:** Service de Réanimation polyvalente et surveillance continue, Assistance Publique—Hôpitaux de Paris, Hôpital Antoine Béclère, Université Paris Sud, Clamart, F-92400 France; Institut National de la Santé et de la Recherche Médicale, INSERM U999, Le Plessis Robinson, F-92060 France

**Keywords:** Critical care, Defecation, Outcome

## Abstract

In a previous retrospective work, it has been postulated that early enteral nutrition was a protective factor against late defecation and its subsequent consequences in critically ill patients. We raise concerns about methodological issues limiting the conclusions.

## Findings

We read with great interest the article by Fukuda et al. dealing with the risk factors for late defecation in critical care patients [1]. The results suggest an independent association between late enteral nutrition (i.e., more than 2 days after admission) and late defecation (i.e., 5 days or more after admission). However, some points might require clarification. First, the authors state that the first intervention to promote defection was earlier in the early as compared to late defecation group (3 (2–4) vs. 6 (4–7) days, *p* < 0.001). Nevertheless, they did not include this variable in their univariate and multivariate analysis of factor influencing the delay of defecation because they found out that the interventions was associated with late defecation. It is stated that this was a result of physicians concern of delayed transit. Nevertheless, patients in the early defecation group received intervention much sooner after physician evaluation. Though fewer patients received interventions in the early defecation subgroup, it still represents 50 % of the cohort. Therefore, we would like to underline that the timing of intervention might also have been considered for the multivariate analysis. Second, though not specifically stated, exclusion criteria do not seem to encompass some conditions clearly affecting transit such as pancreatitis, postpartum patients, hypothyroidism, or occlusive syndrome. We wonder if such patients might have been included in the study, therefore potentially impacting the results. Third, the article does not provide clear explanations for the late beginning of enteral nutrition. Although it is stated in the discussion that some regimens of nutrition are usual in the investigating centre, no protocol seemed to be followed, and the reasons of late enteral nutrition beginning might have been influenced by the attending physician own decision and beliefs. Fourth, only 33/282 patients harbored infection, which seems to be unexpectedly low for a 20 bed ICU after a year of study.
