# Erratum to: Comments to: Risk factors for late defecation and its association with the outcomes of critically ill patients: a retrospective observational study

**DOI:** 10.1186/s40560-016-0183-y

**Published:** 2016-09-13

**Authors:** Dominique Prat, Benjamin Sztrymf

**Affiliations:** 1 Service de Réanimation polyvalente et surveillance continue, Assistance Publique—Hôpitaux de Paris, Hôpital Antoine Béclère, Université Paris Sud, Clamart, F-92400 France; 2 Institut National de la Santé et de la Recherche Médicale, INSERM U999, Le Plessis Robinson, F-92060 France

## Erratum

Unfortunately, after publication of this article [1], it was noticed that the author response was not included in the article text. The below text should have been displayed after the section titled ‘Findings’. The original article has also been updated to include this author response.

## Authors’ response

We appreciate Dr. Dominique’s comments regarding our study [2], “Risk factors for late defecation and its association with the outcomes of critically ill patients: a retrospective observational study”.

The issue of “intervention” was discussed among the authors. We have performed multivariable analysis in which we defined early and late intervention as ≤ 3 days and > 3 days after admission, respectively, and included these as explanatory factors. We found that late intervention was a risk factor for late defecation (odds ratio, 3.00, 95 % confidence interval, 1.50 to 6.02, *P* = 0.002). This did not alter our main findings.

However, unlike other factors, such as sedation, late enteral nutrition, and surgery, it is unsurprising that intervention is closely related to the time to defecation because interventions are usually performed in patients with delayed defecation. Accordingly, our statistician recommended excluding “intervention” from the multivariable analysis.

These results also raise the possibility that early intervention could improve the outcome of patients. This may be true, but we are concerned that discussing this issue could be misleading or alter the conclusions. Furthermore, the study was not designed to address this possibility. A prospective study is necessary to investigate the effects of interventions on the outcomes of critically ill patients being treated in an intensive care unit (ICU).

Our patients included two with pancreatitis and one with paralytic ileus. Nevertheless, it is conceivable that the risk factors identified in our study are also associated with late defecation in patients with these or similar illnesses. We think that patients with direct occlusion of the intestine should be excluded, but patients with indirect dysfunction of intestinal transit can be included in studies such as ours.

As Dr. Dominique has suggested, we should report the timing of enteral nutrition. Because the nutrition regimen in our ICU is not strictly timed, each attending physician determines when to administer nutrition based on various factors, such as the stability of vital signs, the presence of bowel sounds, and the gastric residual volume. All of the physicians in our ICU recognize the importance of enteral nutrition.

We also placed patients with a primary source of infection in an “infection” group. Infection was diagnosed based on the patient’s present and past medical history, physical findings, bacterial cultivation, and biochemical and radiographic findings. Careful examination of the source of infection may be associated with the small number of infectious patients included in our study. In addition, because our emergency ICU accepts severe outpatients, only approximately 30 infectious patients are admitted per year. Infectious patients who died or who were discharged < 6 days after ICU admission were excluded from our study.
